# Attitudes pregnant women in Türkiye towards holistic complementary and alternative medicine and influencing factors: a web-based cross-sectional study

**DOI:** 10.1186/s12906-023-04065-x

**Published:** 2023-07-05

**Authors:** Burcu Küçükkaya, Hafsa Kübra Işık

**Affiliations:** 1 Faculty of Health Sciences, Nursing Department, Division of Gynecology and Obstetrics Nursing, Bartın University Rectorate, 74100 Agdaci Campus, Bartın, Türkiye; 2 Faculty of Health Sciences, Midwifery Department, Bayburt University, Bayburt, Türkiye

**Keywords:** Pregnancy, Complementary medicine, Alternative medicine, Attitudes

## Abstract

**Background:**

Pregnant women turn to holistic complementary and alternative medicine to cope with problems associated with the changes they experience during pregnancy. This study aimed to determine the attitudes of pregnant women in Türkiye toward holistic complementary and alternative medicine and influencing factors.

**Methods:**

This cross-sectional exploratory study was carried out between June and November 2022 with a web-based questionnaire distributed via social media and communication platforms. Two hundred and twenty-one pregnant women participated in the study. A "Participant Identification Form" and the "Attitudes towards Holistic Complementary and Alternative Medicine Questionnaire" were used to collect the data. Logistic regression analysis was used to determine correlations between variables and scale scores.

**Results:**

It was determined that 84.2% of the participants had knowledge about traditional and complementary therapies, and 77.8% used traditional and complementary therapies. The participants reported that they preferred faith (77.4%), energy healing (76.9%), massage (75.6%), diet (74.2%), meditation/yoga (62.0%), and herbal (59.7%) traditional and complementary therapies the most, and most of them used these methods to reduce nausea, vomiting, edema, and fatigue during pregnancy. The mean Attitudes towards Holistic Complementary and Alternative Medicine Questionnaire score of the participants was 35.0 (5.04). It was seen that having high school or higher education (*p* < 0.05), having an income more than expenses (*p* < 0.001), having received advice from nurses when having a complaint (*p* < 0.001), having knowledge about traditional and complementary therapies (*p* < 0.001), and being a practitioner who received services of traditional and complementary therapies (*p* < 0.001) were positively associated with the utilization of traditional and complementary therapies.

**Conclusion:**

In this study, it was determined that the attitudes of pregnant women towards holistic complementary and alternative medicine were high. Their personal characteristics, as well as their knowledge and practice of holistic complementary and alternative medicine affected their attitudes towards holistic complementary and alternative medicine. Obstetrics nurses/midwives should actively participate in training programs on traditional and complementary therapies focused on pregnant women.

## Introduction

Pregnancy is a period in which the female body undergoes physiological, psychological, and social changes, to which women need to adapt [1, 2]. While pregnancy is mostly perceived as a source of happiness, maturation, and self-actualization for some women, it may also create hopelessness, worries, and anxiety. Even though pregnancy is a physiological condition, any complication that may occur during pregnancy has the potential to cause serious strain and psychiatric symptoms in the expectant mother [3–5].

Complementary medicine is described as healthcare practices specific to the culture of a country that are not part of the country’s conventional medicine and cannot be fully integrated into the dominant healthcare system [6]. Although various different terms such as alternative medicine or modern medicine have been used for complementary medicine in recent years, the World Health Organization (WHO) has decided that there can be no alternative to medicine, but only therapies complementary to it, and in line with that decision, the term “traditional and complementary medicine” has come to the fore. According to the National Center for Complementary and Integrative Health (NCCIH), complementary and alternative health approaches are defined as a group of diverse medical and healthcare systems, practices, and products that are not considered to be part of conventional or allopathic medicine [7, 8].

When individuals face a health problem, they try to find a solution. In this solution-seeking process, sometimes modern medicine is used, while traditional and complementary therapies come into play at other times [6]. Nowadays, most people resort to traditional and complementary therapies (T&CT) to maintain and improve their health, prevent the occurrence of diseases, heal diseases, complement their ongoing medical treatment, and protect themselves against the undesirable effects of medications. It is believed that this interest will increase even more in the future [9–11].

Women have been reported to resort to T&CT to relieve their pregnancy-related complaints [1, 12]. Studies in the literature have shown that women use T&CT to relieve their pregnancy-related complaints including fatigue, lower back pain, sleep problems, edema, constipation, and nausea [13–20]. Further, it was reported that as a result of their cultural beliefs and because they have easier access to T&CT, pregnant women use T&CT to relieve symptoms not related to pregnancy, improve their overall health and boost their immune system [21]. Moreover, it was stated that pregnant women turn to T&CT because they believe that medical therapies may negatively affect their health and that of their fetus, because they can take part in making decisions about their own health, and because it gives them more autonomy [14, 22].

A study by Frawley et al. (2013) showed that women relied on non-professional information sources such as advice from friends and family when deciding to use T&CT [23]. Several sources suggested that women do not disclose their use of T&CT to their midwives or obstetricians [24, 25]. Women’s use of T&CT without the knowledge or input of a midwife is a serious cause of concern. Lack of communication with health professionals on the use of T&CT is problematic as it can increase risks and undermine the therapeutic relationship [26]. It is also important that OB/GYN nurses and midwives, who play an important role in women’s health and education in the perinatal period, avoid a judgmental attitude toward pregnant women using T&CT. Given the ever-increasing popularity of T&CT, OB/GYN nurses and midwives are responsible for raising the awareness levels of pregnant women.

There is a significant lack of research on the safety and efficacy of T&CT during pregnancy [12, 24]. The gaps observed in the literature are in-depth investigations of women’s attitudes toward complementary and alternative therapies and influencing factors. The widespread use of T&CT and differences in the types of T&CT used in different cultures laid the groundwork for this study. In this context, it was observed that even though there are studies examining the effects of specific complementary and alternative therapy methods, there are no studies that examine the subject both nationally and internationally. Our aim in conducting this study was to examine the attitudes of pregnant women toward holistic T&CT and influencing factors. The findings of our study will contribute to the relevant literature.

## Methods

### Research design

This cross‐sectional exploratory study was conducted using a web‐based survey sent via pregnancy groups on Facebook, Instagram, or WhatsApp between June and November 2022.

### Participants

**Fig. 1:**
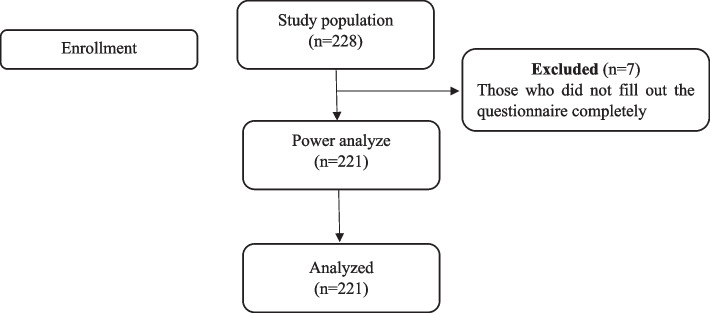
Study flowchart

The convenience sampling method was used in this study to select participants. This method involves a researcher selecting potential respondents based solely on the convenience of their access to them [27]. In the power analysis that was conducted to determine the minimum required sample size of this study, it was determined that the sample needed to include 221 participants based on the study conducted by Özer et al. (2018) in a different sample in the Turkish population that used the Attitudes towards Holistic Complementary and Alternative Medicine Questionnaire (HCAMQ) for the effect size, with a 5% margin of error, 95% confidence interval, 80% power, and 0.05 significance [11]. The inclusion criteria included being pregnant women who were over 18 years old, the ability to speak Turkish, being literate in Turkish, using web applications (Facebook, Instagram, or WhatsApp), being registered to pregnancy groups on social media, and agreeing to participate in the study. The exclusion criteria were being pregnant women who were over 49 years old and not providing consent to participate in the study. All participants who met the inclusion criteria were invited to the study, and the study was conducted with volunteers (Fig. 1).

### Data collection

Pregnant women in pregnancy groups on Facebook, Instagram, or WhatsApp were invited to participate in the study via an online survey link. They were informed about the study on the first page of the online survey. Currently, there are no standardized survey tools suitable to assess the use of traditional medicine among pregnant women in Türkiye. Therefore, this study was based on a new survey tool developed by the authors based on a comprehensive literature review. A researcher who develops a new measure should establish that it has “face validity” as a minimum requirement and that the new measure reflects the content of the concept in question [28]. As an essentially intuitive process, the face validity of the tool used in this study was ensured by using a mixed-methods sequential explanatory design which allowed the triangulation of quantitative and qualitative data on the same topic. The use of a mixed-methods approach assured the validation of the findings of the survey through semi-structured in-depth interviews [29]. Additionally, a pilot test performed before the main analyses to ensure the face validity of the survey (sociodemographic and obstetric characteristics, evaluation of characteristics related to traditional and complementary medicine). The survey consisted of three parts: the first part included questions on sociodemographic and obstetric characteristics, the second part included the evaluation of characteristics related to traditional and complementary medicine, and the third part consisted of 11 items of the Attitudes towards Holistic Complementary and Alternative Medicine Questionnaire (HCAMQ) for evaluating the attitudes of the participants towards T&CT.

### Part 1: evaluation of sociodemographic and obstetric characteristics

Sociodemographic and obstetric data included age, gestational week, number of pregnancies, number of living children, education level, status of working during pregnancy, place of residence, education level of partner, employment status of partner, presence of a chronic illness, status of regular physical activity, status of smoking during pregnancy, general health status, the first source of advice when one has a complaint, sources of advice in case of a complaint, status of having a planned pregnancy, emotional state during pregnancy, status of attending follow-ups during pregnancy, number of follow-ups attended during pregnancy, breastfeeding experience, and experience of education about breastfeeding [11, 13, 15].

### Part 2: evaluation of characteristics related to traditional and complementary medicine

The data collected in this part included knowledge of T&CT, use T&CT, presence of a practitioner who provides T&CT services in close circle, presence of individuals using T&CT in the family, status of obtaining information about T&CT from health personnel, the T&CT method that is used, reason for using T&CT, status of telling health personnel about the T&CT method to be applied, reason for not telling health personnel about the T&CT to be applied, recommendation of T&CT, current and past use status, thinking of using T&CT in the future, and not thinking of using T&CT [10, 13, 15].

### Part 3: attitudes towards holistic complementary and alternative medicine

HCAMQ consists of 11 items and has been previously validated [30, 31]. It was used to evaluate the attitudes of the participants towards T&CT in this study. The measurements are scored according a 6-point Likert-type scale (1 = I absolutely agree, 6 = I absolutely disagree). A minimum of 11 and a maximum of 66 points can be obtained from the scale. Lower scores indicate more positive attitudes towards T&CT. The Cronbach's alpha coefficient of HCAMQ was found to be 0.72, indicating good reliability [31].

### Ethics statement

The Trakya University Scientific Research Ethics Committee (TUTF-GOBAEK 2022/294) approved this study. An informed consent option was presented on the first page of the online survey. All participants were electronically informed on the first page of the survey that they were volunteering to participate and that they could leave the survey without completion at any time. The study was conducted in accordance with the principles of the Declaration of Helsinki.

### Statistical analysis

The normality of the distribution of the data was evaluated with the Kolmogorov–Smirnov test. Descriptive statistics (mean, standard deviation, frequency, percentage, minimum, and maximum) were calculated, and the Mann–Whitney U test, the Kruskal–Wallis test, and Student’s t-test were conducted for determining the significance of the differences between the scale scores of the participants based on their descriptive characteristics. Logistic regression analysis was used to determine predictive relationships between the scale scores of the participants and other variables. The analyses were performed using IBM SPSS Statistics for Windows, Version 23.0 (IBM Corp). *p* < 0.05 was considered statistically significant.

## Results

The demographic, obstetric, and health-related characteristics of the participants are shown in Table 1. The mean age of the participants was 29.2 ± 5.3, and their mean gestational week was 25.5 ± 8.0. It was determined that most participants had high school or higher degrees, 55.7% of the participants considered themselves very healthy, and 85.1% received advice from a doctor in case of a complaint.

**Table 1:** Demografic, obstetric and health related characteristic of pregnant (*n* = 221)

Variables	$\overline{{\varvec{X}} }$ ± SD		
Age (year)	29.2 ± 5.3 (min:18 – max:42)		
Gestation week	25.5 ± 8.0 (min:11 – max:40)		
		***n***	**%**
Educational status	Middle school and below	13	5.9
	High school or higher	208	94.1
Occupation in pregnancy	Working	96	43.4
	Not working	105	47.6
	Maternity leave	20	9.0
Living Place	Province	199	90.0
	Town / Village	22	10.0
Family type	Nuclear family	202	91.4
	Extended family	19	8.6
Partner's educational status	Middle school and below	4	1.8
	High school or higher	217	98.2
Partner's occupation	Working	220	99.5
	Not working	1	0.5
Physical exercise status	Doing	179	81.0
	Not Doing	42	19.0
Smoking Status	Using	21	9.5
	Not using	200	90.5
Presence of chronic disease	Yes	9	4.1
	No	212	95.9
General health status	Too bad	3	1.4
	Bad	4	1.8
	Middle	50	22.6
	Good	41	18.6
	Very good	123	55.7
The first person you get advice from if you have a complaint	Doctor	188	85.1
	Nurse	7	3.2
	Family	26	11.8
Resources from which you can gather advice in case you have a complaint	Television	9	4.1
	Internet	212	95.9
Number of pregnancies	1	143	64.7
	2	55	24.9
	3	18	8.1
	4	3	1.4
	5	2	0.9
Number of children	0	139	62.9
	1	62	28.1
	2	18	8.1
	3	2	0.9
Planned pregnancy status	Yes	192	86.9
	No	29	13.1
Emotional state of pregnancy	Positive	204	92.3
	Negative	17	7.7
Check-up status during pregnancy	Yes	203	91.9
	No	18	8.1
Number of checkups during pregnancy	Did not go	18	8.1
	1–15 times	200	90.5
	Monthly	3	1.4
Breastfeeding experience	Yes	79	35.7
	No	142	64.3
Education about breastfeeding	Yes	185	83.7
	No	36	16.3

$\overline{X }$ Mean, *SD* Standard deviation, *min* Minimum, *max* Maximum

Table 2 presents the characteristics of the participants T&CT. It was determined that 84.2% of the participants had knowledge about T&CT, 77.8% used T&CT, and 71% were receive T&CT service from an expert healthcare professional. It was observed that most of the participants used T&CT in their family, and most received information from health personnel about T&CT. While 82.4% of the participants told health personnel about the T&CT method they applied or would apply, 14% stated that they did not want to tell health personnel about it as they thought they did not need to (Table 2).

**Table 2:** Characteristics of pregnant women regarding traditional and complementary therapy (T&CT) (*n* = 221)

**Variables**		***n***	**%**
Knowledge of T&CT	Yes	186	84.2
	No	35	15.8
The use of T&CT	Yes	172	77.8
	No	49	22.2
If you have received T&CT application service, the presence of healthcare professionals who are experts in the field of T&CT	Yes	157	71.0
	No	64	29.0
Presence of individuals using T&CT practices in the family	Yes	10	4.5
	No	211	95.5
The state of getting information about T&CT from health personnel	Yes	159	71.9
	No	62	28.1
T&CT method used	Herbal	132	59.7
	Diet	164	74.2
	Food	105	47.5
	Vitamin	106	48.0
	Belief	171	77.4
	Leech	4	1.8
Reason for using T&CT	For therapeutic purposes	6	2.7
	Reducing nausea and vomiting in pregnancy	167	75.6
	Reducing edema in pregnancy	161	72.9
	Increasing sleep time during pregnancy	158	71.5
	Reducing fatigue in pregnancy	161	72.9
	Treatment supportive	6	2.7
	Hearing that it is beneficial from the environment	63	28.5
	Finding alternative treatments safe	36	16.3
State of telling the T&CT method applied/considered to be applied to the health personnel	Yes	182	82.4
	No	39	17.6
The reason for not telling the health personnel about the T&CT method applied/considered to be applied	Disapproval	4	1.8
	Negative reaction	4	1.8
	Not needing	31	14.0
Recommendation of T&CT methods	Yes	185	83.7
	No	36	16.3

The HCAMQ scores of the participants are shown in Table 3. The mean total HCAMQ score of the participants was 35.0 ± 4.4 (min:11 – max:46).

**Table 3:** Results of the attitudes towards holistic complementary and alternative medicine of pregnant women (*n* = 221)

Variables	$\overline{{\varvec{X}} }$± SD
Holistic Complementary and Alternative Medicine Questionnaire (HCAMQ)	35.0 ± 4.4 (min:11 – max:46)

$\overline{X }$ mean, *SD* standard deviation, *min* minimum, *max* maximum

Table 4 shows the results of the comparisons of the personal, obstetric, and T&CT-related characteristics of the participants and the descriptions of their attitudes towards T&CT. It was determined that the participants who had high school or higher education, those who had a higher income than their expenses, those who had nuclear families, those who had a spouse's education level of high school or higher education, those who did not smoke, those who used vitamins, those with higher numbers of pregnancies and children, those who attended pregnancy follow-ups, those who had breastfeeding experience, those who had received no education about breastfeeding, those who used T&CT, receive T&CT service from an expert healthcare professional, those using T&CT practices in their families, those who were following a diet, those using faith-related methods, those using T&CT for therapeutic purposes, those using T&CT to reduce nausea and vomiting during pregnancy, reduce edema during pregnancy, increase their sleep duration during pregnancy, alleviate fatigue during pregnancy, and as a supplementary treatment, and those who explained the T&CT method they considered to health personnel had more positive attitudes towards T&CT (*p* < 0.05).

**Table 4:** Comparison of pregnant women's personal, obstetric, traditional and complementary medicine characteristics and their level of attitude towards HCAMQ

Variables		HCAMQ	
		***t***	***p* Value**
Age (year)		-12.507	** < .001**^**T**^
Gestation week		-14.545	** < .001**^**T**^
		$\overline{{\varvec{X}} }$**± *SD***	
Educational status	Middle school and below	39.0 ± 4.4 (min:33 – max:46)	**.012**^**U**^
	High school or higher	34.8 ± 4.3 (min:11 – max:44)	
Income status	Income less than expenses	37.0 ± 6.5 (min:18 – max:46)	** < .001**^**K**^
	Income equals expense	35.3 ± 3.0 (min:19 – max:41)	
	Income more than expenses	27.5 ± 8.3 (min:11 – max:36)	
Family type	Nuclear family	34.7 ± 4.2 (min:11 – max:41)	** < .001**^**U**^
	Extended family	38.8 ± 4.9 (min:27 – max:46)	
Spouse's educational status	Middle school and below	35.0 ± 4.2 (min:11 – max:44)	**.012**^**U**^
	High school or higher	34.5 ± 12.2 (min:18 – max:46)	
Smoking status	Using	38.6 ± 3.8 (min:33 – max:46)	** < .001**^**U**^
	Not using	34.7 ± 4.3 (min:11 – max:44)	
Vitamin usage status	Using	19.5 ± 12.0 (min:11 – max:28)	**.005**^**U**^
	Not using	35.2 ± 4.1 (min:16 – max:46)	
Number of pregnancies	1	35.4 ± 4.5 (min:11 – max:46)	**.010**^**K**^
	2	34.3 ± 4.3 (min:19 – max:41)	
	3	36.2 ± 2.1 (min:31 – max:41)	
	4	30.0 ± 7.9 (min:21 – max:36)	
	5	30.5 ± 2.1 (min:29 – max:32)	
Number of children	0	35.8 ± 3.8 (min:16 – max:46)	**.001**^**K**^
	1	34.1 ± 4.7 (min:11 – max:41)	
	2	33.6 ± 5.8 (min:19 – max:41)	
	3	26.5 ± 7.8 (min:21 – max:32)	
Check-up status during pregnancy	Yes	34.7 ± 4.2 (min:11 – max:41)	** < .001**^**U**^
	No	39.0 ± 4.3 (min:33 – max:46)	
Breastfeeding experience	Yes	34.2 ± 4.2 (min:19 – max:41)	**.004**^**U**^
	No	35.5 ± 4.4 (min:11 – max:46)	
Education about breastfeeding	Yes	35.4 ± 3.2 (min:16 – max:41)	**.003**^**U**^
	No	33.1 ± 7.8 (min:11 – max:46)	
The use of T&CT	Yes	32.3 ± 8.2 (min:11 – max:46)	** < .001**^**U**^
	No	35.8 ± 1.8 (min:23 – max:41)	
The presence of a practitioner who receives T&CT practice services	Yes	32.8 ± 7.4 (min:11 – max:46)	** < .001**^**U**^
	No	36.0 ± 1.5 (min:24 – max:41)	
Presence of individuals using T&CT practices in the family	Yes	32.7 ± 2.8 (min:28 – max:36)	** < .001**^**U**^
	No	35.1 ± 4.4 (min:11 – max:46)	
T&CT method used/Diet	Yes	32.8 ± 7.7 (min:11 – max:46)	**.004**^**U**^
	No	35.8 ± 1.8 (min:23 – max:41)	
T&CT method used/Belief	Yes	32.3 ± 8.2 (min:11 – max:46)	** < .001**^**U**^
	No	35.8 ± 1.7 (min:25 – max:41)	
Reason for using T&CT/For therapeutic purposes	Yes	27.3 ± 5.5 (min:19 – max:36)	** < .001**^**U**^
	No	35.2 ± 4.2 (min:11 – max:46)	
Reason for using T&CT/Reducing nausea and vomiting in pregnancy	Yes	33.0 ± 7.8 (min:11 – max:46)	**.017**^**U**^
	No	35.7 ± 2.1 (min:21 – max:41)	
Reason for using T&CT/Reducing edema in pregnancy	Yes	32.7 ± 7.5 (min:11 – max:46)	** < .001**^**U**^
	No	35.9 ± 1.7 (min:22 – max:41)	
Reason for using T&CT/Increasing sleep time during pregnancy	Yes	32.7 ± 7.4 (min:11 – max:46)	** < .001**^**U**^
	No	35.9 ± 1.5 (min:23 – max:41)	
Reason for using T&CT/Reducing fatigue in pregnancy	Yes	32.8 ± 7.5 (min:11 – max:46)	** < .001**^**U**^
	No	35.9 ± 1.8 (min:23 – max:41)	
Reason for using T&CT/Treatment supportive	Yes	27.5 ± 9.3 (min:11 – max:36)	** < .001**^**U**^
	No	35.2 ± 4.0 (min:16 – max:46)	
State of telling the T&CT method applied/considered to be applied to the health personnel	Yes	33.3 ± 7.3 (min:11 – max:46)	**.009**^**U**^
	No	35.7 ± 2.1 (min:23 – max:41)	
The reason for not telling the health personnel about the T&CT method applied / considered to be applied	Yes	31.4 ± 8.2 (min:11 – max:44)	** < .001**^**U**^
	No	35.8 ± 2.4 (min:23 – max:46)	

$\overline{X }$ mean, *SD* standard deviation, *min* minimum, *max* maximum

^T^Student T-test

^U^Mann Whitney U, and ^K^Kruskall Wallis

The results of the comparisons of the HCAMQ scores of the participants based on the T&CT that they were currently using are shown in Table 5. A significant relationship was found between the T&CT methods currently used by the participant (meditation / yoga, Tai chi, chiropractic methods, homeopathy, cupping, massage, reflexology, energy healing, lifestyle diets) and their HCAMQ scores (*p* < 0.05).

**Table 5:** Comparison of the level of attitudes towards HCAMQ with T&CT currently used by pregnant women

**Variables**			**HCAMQ**	
		***n* (%)**	$\overline{{\varvec{X}} }$**± *SD***	***p* Value**
Meditation/Yoga	Yes	137 (62.0)	33.7 ± 6.6 (min:11 – max:46)	**.014**^**U**^
	No	84 (38.0)	35.8 ± 1.8 (min:22 – max:41)	
Taichi	Yes	2 (0.9)	28.0 ± 0.0 (min:28 – max:28)	**.009**^**U**^
	No	219 (99.1)	35.1 ± 4.4 (min:11 – max:46)	
Karyopractic	Yes	2 (0.9)	20.5 ± 3.5 (min:18 – max:23)	**.003**^**U**^
	No	219 (99.1)	35.2 ± 4.2 (min:11 – max:46)	
Homeopathy	Yes	4 (1.8)	31.0 ± 5.7 (min:23 – max:36)	**.013**^**U**^
	No	217 (98.2)	35.1 ± 4.3 (min:11 – max:46)	
Cupping	Yes	1 (0.5)	21.0 ± 0.0 (min:21 – max:21)	**.039**^**U**^
	No	220 (99.5)	35.1 ± 4.3 (min:11 – max:46)	
Massage	Yes	167 (75.6)	33.0 ± 7.5 (min:11 – max:46)	**.003**^**U**^
	No	54 (24.4)	35.7 ± 2.4 (min:16 – max:41)	
Reflexology	Yes	12 (5.4)	33.1 ± 4.1 (min:23 – max:36)	**.008**^**U**^
	No	209 (94.6)	35.1 ± 4.4 (min:11 – max:46)	
Energy Healing	Yes	170 (76.9)	32.4 ± 7.9 (min:11 – max:46)	** < .001**^**U**^
	No	51 (23.1)	35.8 ± 2.0 (min:18 – max:41)	
Lifestyle diets	Yes	170 (76.9)	32.5 ± 8.0 (min:11 – max:46)	**.001**^**U**^
	No	51 (23.1)	35.8 ± 1.9 (min:21 – max:41)	

$\overline{X }$ mean, *SD* standard deviation, *min* minimum, *max* maximum

^U^Mann Whitney U

The results of the logistic regression analysis showing factors affecting the use of T&CT among the participants are presented in Table 6. The participants who had received advice from a physician when they had a complaint were 52.389 times more likely to use T&CT compared to those who had received advice from nurses and family (p < 0.001). The participants with nuclear families were 45.156 times more likely to use T&CT compared to those with extended families (p < 0.001). The participants with higher income and educational levels were 8.268 and 12.225 times more likely to use T&CT compared to those with lower income (*p* < 0.001) and educational status (*p* < 0.05). Being on maternity leave (*p* < 0.001), not doing physical exercise (*p* < 0.001), being a smoker (p < 0.01), perceiving one’s general health to be very good (*p* < 0.001), having knowledge about T&CT (*p* < 0.001), and receive T&CT service from an expert healthcare professional (*p* < 0.001) were positively associated with the utilization of T&CT.

**Table 6:** Logistic regression analysis for determining factors affecting T&CT use in pregnant

Characteristic	Group	OR	95% Cl		*p* Value
Education	Middle school and below	12.225	1.925	77.630	**.008**
	High school or higher				
Income status	Income less than expenses	8.268	2.554	26.762	** < .001**
	Income equals expense				
	Income more than expenses				
Family type	Nuclear family	45.156	9.945	205.036	** < .001**
	Extended family				
Working status during pregnancy	Working	4.550	1.102	18.788	** < .001**
	Not working				
	Maternity leave				
Physical Exercise Status	Doing	.078	.036	.169	** < .001**
	Not doing				
Smoking Status	Using	16.194	5.547	47.275	** < .001**
	Not using				
General health status	Too bad	0.171	.68	.426	** < .001**
	Bad				
	Middle				
	Good				
	Very good				
The first person you get advice from if you have a complaint	Doctor	52.389	14.608	187.879	** < .001**
	Nurse				
	Family				
Knowledge of traditional and complementary therapies	Yes	.003	.000	.020	** < .001**
	No				
Presence of a practitioner who receives traditional and complementary therapy service	Yes	.005	.001	.021	** < .001**
	No				

## Discussion

Traditional, complementary and alternative medicine (T&C) has become increasingly popular among pregnant women all over the world. Recent research showed that more than one-third of pregnant women in the United States used one or more T&CT in the previous year [32, 33].

Our study showed that the rate of using at least one T&CT during pregnancy was 77.8%. In a study conducted in the United Kingdom, it was found that 57.1% of women used at least one T&CT during pregnancy [18], and this rate was 91.5% in Palestine [34], 56.7% in Iraq [32], 85.2% in Malaysia [35], 50.7% in Kenya [36], and 56.92% in Iran [21]. In the relevant literature, it is seen that the use of T&CT has differed from country to country [37–39]. It is thought that these differences in results are due to sample dynamics and sociodemographic differences.

Another important result of this study was that 71.9% of the participants had received information about T&CT from healthcare personnel. Moreover, 82.4% of the participants stated that they would share the T&CT method they would apply with health personnel. Additionally, it was determined that whether the participants received information about T&CT and whether they would tell health personnel about the T&CT method to be applied also affected their attitudes towards T&CT. In previous studies, it was stated that pregnant women could not get enough information about T&CT because they could not communicate with health personnel [31, 40]. In this sense, health personnel need to be impartial and non-judgmental to facilitate communication and encourage women to tell them about the methods they use. Furthermore, it is thought that the knowledge and attitudes of health personnel about T&CT affect pregnant women's decisions to use T&CT and the T&CT method they use. Nonetheless, more research is needed on this topic.

In our study, a significant relationship was determined between the reasons underlying the decisions of the participants to use T&CT and their attitudes toward T&CT. In Western countries, it has been observed that pregnant women use T&CT to treat their pregnancy-related physical symptoms and complaints [39, 41–44]. In our study, it was found that the participants mostly used T&CT to relieve nausea, vomiting, edema, and fatigue during pregnancy and increase their sleep duration. Adams et al. (2011) reported that T&CT was used as a means to prevent pregnancy-related complaints [45]. Other previous studies have also shown that pregnant women resorted to T&CT to seek relief for pregnancy-related complaints like nausea, lower back pain, pelvic girdle pain, headache, and migraine, improve sleep quality, and reduce fatigue [23, 39, 43, 46, 47].

In the literature, pregnant women’s concerns about the side effects of medications and their beliefs that T&CT are more effective than medical treatment have been reported to be among their reasons for using T&CT [34, 48–50]. In another study, it was determined that pregnant women used T&CT to keep control over their bodies and lives and have an unmedicated natural delivery, and they applied these therapies actively and autonomously [22, 51]. In this context, it is thought that the differences observed in the types of T&CT used by women to relieve their pregnancy-related complaints are attributable to differences in traditional cultural/religious beliefs and geographical characteristics. To eradicate misconceptions about the safety of T&CT in pregnant women, it is advisable to conduct evidence-based studies that will demonstrate the advantages and disadvantages of these therapies.

According to the results of our study, the T&CT used the most frequently by the participants were meditation/yoga, Tai chi, chiropractic methods, homeopathy, cupping, massage, reflexology, energy healing, and lifestyle diets. It was found that the differences in the attitudes of the participants towards T&CT based on the types of therapy they used were significant. It has been observed in other studies that massage, yoga, and meditation are the most common therapies used during pregnancy, and these results are similar to the findings of our study [25, 34, 43, 51–53]. Along with these results, studies conducted in different countries have reported that homeopathy, massage, herbs, and vitamins are also used [32, 33, 48, 54]. In this sense, it is thought that regional differences, differences in culture and tradition, and the provision and availability of T&CT services by region are the reasons for differences seen in the types of T&CT used by women during pregnancy.

In our study, the significant predictors affecting T&CT use in the participants were being a person with a high school or higher degree, having an income greater than expenses, having a nuclear family, being on maternity leave, not performing physical exercises, being a smoker, perceiving one’s general health to be very good, getting advice from a nurse in the case of having a complaint, having knowledge about T&CT, and having access to a T&CT practitioner. Studies have shown that women’s educational statuses are among the statistically significant predictors of T&CT use [23, 25, 37, 43, 48, 50]. It is considered that when pregnant women have higher education levels and income levels and when they are employed, their awareness will grow, and hence, the they will learn about and have more positive attitudes toward T&CT.

In our study, being a smoker and lack of physical exercise were among the significant predictors T&CT usage, suggesting that the motivation behind women’s use of T&CT is to make healthy lifestyle changes instead of quitting harmful habits. Another significant predictor affecting the usage of T&CT among the participants was having knowledge about T&CT, while no indication of this important finding could be found in the literature. Considering that the other significant predictors in our study were getting advice from a nurse in the case of having a complaint and having access to a practitioner, it can be concluded that pregnant women feel comfortable sharing the T&CT therapies they use with their healthcare providers.

The results of our study and other studies in the relevant literature suggest that the prevalent usage of T&CT during pregnancy constitutes an important concern for the protection and improvement of public health. Due to the lack of evidence-based studies on the use of T&CT during pregnancy, their reliability in terms of maternal and fetal health remains unknown. Health professionals should question the use of T&CT by pregnant women and create an environment that will encourage the disclosure of these methods by them. In this sense, while communicating effectively on the subject, health professionals should have evidence-based knowledge of T&CT, and they should guide pregnant women about the benefits and risks it can bring.

### Strengths and limitations

One of the major strengths of this study is that it explored the factors affecting T&CT use by revealing the attitudes of pregnant women towards T&CT use. Accordingly, it provides information about the important points that health professionals will evaluate during pregnancy by determining the main factors that lead pregnant women to use T&CT. Additionally, it was revealed that pregnant women living in Türkiye have traditional and cultural beliefs associated with T&CT use.

Although the cross-sectional design highlights the connotations of attitudes towards T&CT use, implications for the causality of T&CT use among respondents remain limited. Indeed, knowledge of reasons for T&CT use will improve the interpretation of the relationships of personal, obstetric, and traditional and complementary therapy-related factors with T&CT use among pregnant women. Additionally, the study was conducted with the participation of pregnant women using web applications (Instagram, Facebook, and WhatsApp) in Türkiye. For this reason, the presence of traditional, social, cultural, and economic differences according to regions throughout the country does not allow the generalization of the results of this study to the general population. Finally, although the self-reported use of T&CT takes into account use during pregnancy, recall bias may still have affected the responses of the participants. The authors argue that provider-based T&CT would provide realistic reporting, as these modalities require discussion with the provider and may be easier to remember, but the under-reporting of non-provide-based/self-managed T&CT is likely.

### Implications of the study

Our findings add to the existing literature on the use of T&CT among pregnant women in Türkiye and reveal factors associated with the use of T&CT. Obstetrics nurses and midwives, who are important members of healthcare teams, should be therapeutically unbiased about the reasons for and statuses of using T&CT in their interactions with pregnant women and should examine the factors associated with the use of T&CT in detail. If obstetrics nurses and midwives provide clear and comprehensible communication to pregnant women about T&CT, they will begin to identify the reasons and associated factors that lead them to use T&CT and encourage them to talk about these methods. This may also help discourage the use of T&CT methods whose level of evidence has not been established, and whose safety is questionable. Additionally, this can also guide regulators in the continued development of nursing and midwifery education programs for professionals as well as the health equity of care programs for pregnant women. Since the participants of this study were pregnant, there is a need for similar studies among different samples. Understanding the clinical factors associated with the use of T&CT among pregnant women may reveal deficiencies in the conventional healthcare system while strengthening nursing care.

## Conclusion

The results of this study indicated that most pregnant women living in Türkiye used T&CT. Additionally, use of T&CT among pregnant women was associated with personal, obstetric, and T&CT-related factors. In the study, it was observed that the participants mostly used lifestyle diets and energy healing methods, followed by massage and meditation/yoga. The limited number of studies evaluating the efficacy and safety of T&CT methods in the literature has revealed the importance of this study. These results should serve as a reminder to conduct qualitative studies that reveal cultural and geographical differences in terms of motivating factors in the use of T&CT during pregnancy, and obstetrics nurses and midwives should be actively involved in education programs on this topic. Consequently, although the use of T&CT in different samples differs in different geographical regions, it may be recommended to conduct an international multi-center study evaluating T&CT methods in the future, especially in countries with similar healthcare systems.

## Data Availability

The datasets used and/or analyzed in this study are not publicly available due to the sensitive nature of the interviews. For requests of the data of this study, the corresponding author should be contacted.

## Abbreviations

WHO: World Health Organization
T&CT: Traditional and Complementary Therapies
HCAMQ: Holistic Complementary and Alternative Medicine Questionnaire
NCCIH: National Center for Complementary and Integrative Health
SD: Standard Deviation
